# The maturity of lean management in a large academic medical center in Finland: a qualitative study

**DOI:** 10.1093/intqhc/mzae111

**Published:** 2024-12-10

**Authors:** Irmeli Hirvelä, Paulus Torkki, Mervi Javanainen, Elina Reponen

**Affiliations:** Head and Neck Center, Helsinki University Hospital, Helsinki 00290, Finland; Department of Public Health, Faculty of Medicine, University of Helsinki, Helsinki 00014, Finland; Abdominal Center, Helsinki University Hospital, Helsinki 00290, Finland; Shared Group Services, Gustomer Relations, Helsinkin University Hospital, Helsinki 00290, Finland

**Keywords:** Lean management, maturity, healthcare leader, qualitative study

## Abstract

**Background:**

Lean management (LM) provides hospitals with tools to respond to today’s rapidly changing healthcare environment. However, evidence of its success is inconclusive. In some cases, well-executed LM supports effective, beneficial, and safe patient care; reduces costs; and increases patient and staff satisfaction. In other cases, however, the desired outcomes have not been achieved. Organizations must acknowledge the maturity level of LM to successfully implement it for continuous development. This study evaluates the maturity of Lean implementation using a structured interview with a framework based on the Lean Healthcare Implementation Self-Assessment Instrument (LHISI) and utilizes findings about Lean adoption to evaluate factors that support and hinder its implementation, with the aim of assisting leaders in maintaining and developing Lean in health care.

**Methods:**

The article describes a case study done at Helsinki University Hospital. A qualitative study was conducted in three sectors (A, B, and C) of the hospital. Fifteen healthcare leaders from the three sectors participated in a semistructured interview based on the dimensions of the LHISI. Qualitative content analyses were based on grounded theory.

**Results:**

We concluded that the five dimensions (leadership, commitment, standard work, communication, and daily management system) of LHISI provide a comprehensive framework for qualitatively evaluating Lean in the hospital. We found that the five dimensions are influenced by other explanatory factors. These explanatory factors, knowledge about Lean, available data, and environmental, psychological, and organizational factors all support and hinder leadership, communication, daily management, and commitment to Lean in the hospital. The results highlight differences in the Lean maturity levels in the hospital. We noticed that 9 of 15 leaders had a misunderstanding of Lean, and all 3 sectors showed a lack of staff commitment to Lean in their units.

**Conclusion:**

To strengthen the organization-wide implementation of Lean, it is necessary to understand that LM is a comprehensive sociotechnical management system, for which it is not enough to mechanically implement Lean with tools and techniques alone. By focusing on and developing the five dimensions and explanatory factors, organizations can achieve a high maturity of Lean and reach their full potential. A good level of competency and commitment to Lean by the leaders and the staff alike are important for achieving goals, engaging the staff, and increasing the quality of patient care in the hospital. The long-term Lean development of a hospital organization can be followed and continuously maintained via easy-to-use maturity tools.

## Introduction

Globally, health care exists in an ever-changing and multiform environment, prompting all leaders to think about how to best respond quickly and effectively to customer needs with lower costs and high quality of care. The competition for skilled employees is fierce, meaning that healthcare leaders must think about how to make organizations more attractive. Lean management (LM) has been implemented in many organizations as a sociotechnical system that emphasizes staff empowerment and continuous improvement of the organization to achieve effective performance with low costs, high quality of care, and good job satisfaction [1–5].

LM originated in the Toyota automobile factory in 1954 [6], and it is now widely practiced in the manufacturing and service sectors [1, 7]. In the 2000s, LM was integrated into the health care to promote a culture of continuous improvement and systematic waste reduction [1–4]. LM contributes to developments in the organizational culture of healthcare facilities in such a way that improvement activities are employee-oriented and integrated as a part of their everyday work routines [8]. However, research findings on Lean are still mixed because the implementation of Lean has failed or has been superficial in some organizations [3, 9, 10] and most of the published studies have been based on case studies with weak empirical evidence [1, 7, 10]. A study of an organization-wide analysis is lacking a qualitative research framework for assessing Lean maturity. Therefore, we focus on literature considering the maturity assessment and evaluation of Lean implementation.

To better understand, integrate, and maintain the depth of Lean, maturity assessment tools and methods have been created to help leaders to follow an organization-wide Lean development and identify factors that influence implementation. Still, most of these Lean assessment tools, models, and methods have limitations in health care, as those were developed for industry or service sectors [11]. In addition, some assessment tools are mathematically complex and do not consider all Lean principles [12], which makes such tools difficult to apply and validate [11]. Some tools are only used by external consultants and researchers rather than hospital staff [9]. These types of limitations make it difficult for leaders and staff to use the maturity tools, methods, and models in practice [9, 13] and find factors that support and hinder Lean implementation.

For example, in health care, certain Lean maturity models assess Lean through a qualitative approach [1, 14–16], while other methods combine theoretical and empirical insights [17]. Malmbrand and Åhlström [17] developed a maturity tool in a European context consisting of 16 items to measure Lean maturity in the service sector that makes it possible to focus on the customer/patient in health care. Kaltenbrunner *et al*. [13] refined Malmbrand’s maturity tool in the US health care. This tool, called the Lean in Healthcare Questionnaire, is based on Likert’s Lean description [12] and consists of 16 items, including a measure of staff satisfaction [13]. Marsilio *et al*. [18] compared the Lean maturity of two international hospitals by using The National Survey of Lean tool in the USA and Italy. Roszell [19] created a survey for nurses, but it consists of more than 110 items, making it difficult to use on a regular basis. Charns *et al*. [20] applied multivalue coincidence analysis to interviews with 121 leaders in research and scored Lean markers of transformation. Researchers have likewise identified the following enablers of Lean change: management support, a plan for Lean develop, and two-way communication. They could not, however, connect Lean to outcomes at hospitals [20]. Kunnen *et al*. [21] found that a fruitful organizational and personnel learning culture supports LM implementation, while overloading employees, a lack of commitment, resources, project tracking, and weak knowledge of LM among leaders hinder LM implementation. Hilverda *et al*. [22] emphasize the importance of a good leadership style to increase LM maturity. Above all, by using Lean assessment tools, the studies show that there is a strong relationship between Lean maturity, quality of care, staff and patient satisfaction, and performance of hospitals [15, 18, 23].

To provide a better tool to measure the maturity of Lean implementation in hospitals, the Lean Healthcare Implementation Self-Assessment Instrument (LHISI) was developed by the Center for Lean Engagement and Research at the University of California, Berkeley School of Public Health [7]. Reponen *et al*. [9] validated the LHISI maturity tool in a Finnish healthcare context in 2020. They validated the tool by using reviews of experts, practitioners, and leaders; a pilot test; and a survey. The LHISI survey was sent to all 26 172 employees at the Helsinki University Hospital, 6073 of whom responded (the response rate was 27%). The original LHISI maturity tool was reduced from 43 to 25 items and identified the 5 dimensions of leadership, commitment, standard work, communication, and daily management system through factor analysis. The researchers noticed that the LHISI is a practical tool that can assist in monitoring Lean implementation both overall and in individual departments [9]. Currently, the LHISI is being used in the USA, Spain, and China, while Brazil is planning to use it. Our research uses an LHISI framework and its dimensions to evaluate Lean maturity in the hospital through qualitative research.

This study responds to a qualitative research gap on the maturity assessment and evaluation of comprehensive Lean implementation concerning the organization-wide health care. The research compares Lean implementation in selected hospital sectors by using structured interviews according to a dimension-based framework of LHISI. The purpose of this study is to evaluate what kind of maturity difference exists between sectors, and what kind of factor affects Lean implementation and then organization.

## Methods

The research was conducted by interviewing healthcare leaders in a single academic medical center. The interview content was based on the dimensions of the LHISI, and the interviews were conducted and analyzed during the summer of 2022. One researcher, who was trained in Lean methodology and has 6 years of experience as a Lean coach, interviewed all participants.

### Research settings

The research involves a case study done at the large academic center, Helsinki University Hospital, in Finland. Health care at the large academic hospital is mainly publicly organized and maintained with tax revenue. Finland utilizes the tax-based, Beveridge-type healthcare system model, in which the government provides health care for all its citizens. In the Uusimaa region, primary health care is provided in “well-being services counties” established as part of the health care and social services reform of 2022. These well-being services counties purchase specialized health care from the Helsinki University Hospital.

The Helsinki University Hospital is the largest provider of specialized health care in Finland, and its employees are over 27 000 multiprofessional workers. Every year, ∼680 000 people are treated at the hospital. Sectors A, B, and C are presented in Table 1.

**Table 1.** Employees and treated patients in 2022

Year 2022	Sector A	Sector B	Sector C
Employees	1100	1186	1964
Treated patients in the own sector	157 000	473 650	53 800
Treated patients with purchased services	30 000		
Treated patients in other sectors			83 600

### Data collection and analysis

The 15 leaders interviewed were from the 3 sectors and their divisions, with 14 being experienced in LM and trained in Lean methodology. The volunteer healthcare leaders received the interview questions in advance (Appendix, Attachment 1), and they signed a consent-to-be-interviewed form before the interview. The interviews were conducted online using Microsoft Teams or in person. We used semistructured interviews, in which the interviewer asked the interview questions and clarified the questions, as needed. The interviews averaged 1 h and were recorded and transcribed.

We used grounded theory to analyze the text by identifying the main concepts. We used the main concepts related to the phenomenon, which were also identified through the analysis of the material. When analyzing the interview material, we created new subconcepts related to the phenomenon. These subconcepts were created in the analysis part of the material, where we divided the interview material into different parts, recombined them, and compared the different parts of the material looking for differences and similarities. Based on the research data, subconcepts were created under the main concepts, which revealed the complexity of Lean implementation and the related factors [24].

## Results

In our research, we compared three sectors at the Helsinki University Hospital, and we present our findings collectively in Table 2. We used a simple term—leader—to describe the division and sector leaders of the hospital who were interviewed.

**Table 2.** Comparison of the sectors

	Sector A	Sector B	Sector C
Leadership			
How long has Lean been used?	12 years	7 years	10 years
How many Lean coaches are there?	3	Over 20	9
Tiered management system is used	X	X	X
Engage in daily routines in the sector	X	X	X
Engage in daily routines in all divisions	X	X	X
Engage in daily routines in all units		X	
Utilize Lean development tools in daily routines		X	
Commitment			
Commitment at the sector level	X	X	X
Commitment at the division level	X	X	X
Commitment at the unit level			
Evaluation and continuous improvement in the sector	X	X	X
Evaluation and continuous improvement in all divisions	X	X	X
Evaluation and continuous improvement in all units			
Several Lean development tools are used	X	X	X
Peer review is used in units to develop processes		X	
Staff actively generate development proposals		X	
Standard work			
All mandatory work is described, recorded, and standardized		X	
The orientation of a new employee is standardized			
Staff knowledge and abilities are checked based on annual standardized reviews		X	
The ownership of several patient processes has been defined		X	
Regular internal quality tours		X	
Communication			
Standardized meetings and follow-up metrics are used in the sector	X	X	X
Standardized meetings and follow-up metrics are used in all divisions	X	X	X
Standardized meetings and follow-up metrics are used in all units		X	
Common goals are known by all employees			
Daily management system			
Digital daily management boards are used in the sector	X	X	X
Digital daily management boards are used in the divisions	X	X	X
Physical or digital daily management boards are used in all units		X	
Evaluating of effectiveness			
Lean has been useful for meeting the needs of sector/divisions/units	X	X	X
Some successful Lean implementation projects have been completed	X	X	X
Some Lean implementation projects were not completed	X	X	X
Some Lean implementation projects are ongoing	X	X	X
The patients have benefited from Lean implementation	X	X	X
The staff can move around the sector as needed		X	

### Comparing Lean implementation

All 15 leaders described LM as an organization’s culture and philosophy, wherein they were committed to continuously developing and streamlining effective patient healthcare processes. Even though 14 of the 15 leaders had been trained in Lean, there were variations in their knowledge of the Lean philosophy, and they expressed misunderstanding of LM. The misunderstanding was noticeable in the leaders of Sectors A and C. It was evident that nine leaders in the three sectors lacked knowledge about Lean and its implementation strategy was missing. They understood Lean as one development tool for making individual project-like improvements that were affected by other external factors.

In general, it’s just that you have to figure it out somehow, Lean helps you get through it, makes it a structure. And maybe that helps me to see that even though it looks hopeless, when you do things like that from now on. Maybe I see, it in the way that this is one tool, it helps us to move forward, but whether it’s that kind of usability. Is it as good for their development—I feel like it’s not necessarily always the case. (Leader A5)

In all three sectors, the staff commitment to LM varied, which caused problems when trying to implement a Lean culture and development in the units. A primary difference between the three sectors was in how they engaged with LM in their unit, division, and sector routines and how they used Lean development tools. Of the three, Sector B was the most mature sector and its leaders had implemented LM smoothly as part of daily operations; Lean had become part of the sector’s culture and development.

Recently, one of our very experienced nurses said that now she has an idea, how to make one of the small processes go smoothly, so that’s it. That they will come to me, that this is the way it is, that this is the way of thinking, that this is how it is done. Then, when I feel that Lean has remained as a word, especially there in the background, that we just think that how can we be more flow, maybe like this, and then on the other hand, leading with information has become strongly involved. That’s why, there’s a lot on that board, what we’re like and what we do and what takes a lot of time and what costs what and then things like that. (Leader B5)

Sector B had over 20 Lean coaches, who supervised that all development progress was conducted efficiently. The sector’s staff actively generated development proposals and were ready to implement them. Even though the leaders of Sector B did not always have real-time data available, the leaders and the staff were involved in continuous improvement. They had standardized all the mandatory work and shared knowledge about it, and leaders assessed the abilities of the staff on a yearly basis during annual assessment discussions. They recorded the responsibilities for the patient processes, and how they follow up and improve those processes. They had internal quality reviews, wherein issues were discussed with the staff. The staff, leader, Lean coach, and quality control manager attended the reviews. In contrast, all such actions were lacking in Sectors A and C.

All 15 leaders emphasized that the desired results had been achieved in development activities, such as the elimination of waste, maximization of resources (staff, facilities, and time), and streamlining of patient flow. Above all, patients received medical care quickly and their care was safe and standardized at a high quality.

Well, the first thing is that the availability of treatment, i.e. access to treatment, is realized and to some extent the guarantee of treatment is still there. And a large part of that is thanks to Lean, waiting times have been reduced to the turnaround times of the emergency department. The quality of treatment is standardized and from medication to processes. And then through these standards and aftercare guidelines, I would see that it’s beneficial across the hospital. This whole chain of patients and especially now, when the staff turnover is quite high. Then not only the customer base, but also the staff is multinational. And it comes from a wide variety of educational backgrounds that we have fixed acting in a certain way. However, this gives support to the operation as a whole. (Leader B1)

### The factors of Lean implementation

According to our findings, Lean is a sociotechnical management system at the large academic medical center. It includes work or technical systems (work processes and information flow) and social systems: the people and culture surrounding the work processes.

**Figure 1:**
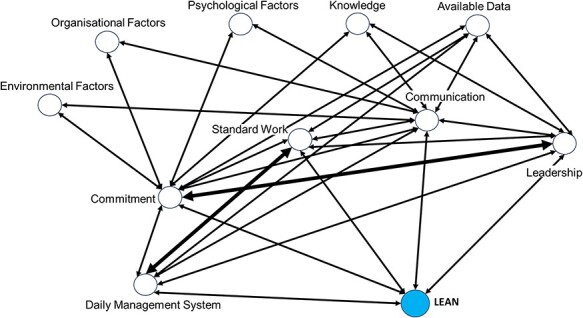
Explanatory factors that strengthen and/or weaken the key factors related to Lean.

The LHISI maturity dimension offers a framework for evaluating the complexity of Lean. Each of the five dimensions is influenced by other explanatory factors. We found that explanatory factors that affect some of the key dimensions influenced, either together or separately, other key dimensions. Figure 1 shows the explanatory factors that strengthen and/or weaken the key dimensions of Lean. The key dimension of leadership influences factor knowledge, which influences communication and commitment. We noted a strong connection between leadership and commitment to Lean. In addition, organizational, psychological, and environmental explanatory factors affect both the key dimensions of communication and commitment. Development influences the key dimensions of commitment and daily management system. We also observed a strong connection between standard work and daily management system. The available data, or lack thereof, affected all the key dimensions.

The factors that support and hinder commitment to LM in the three sectors are presented in Table 3. According to the healthcare leaders, the factors that helped them successfully adopt LM in the different units, divisions, and sectors included organizational and psychological factors, knowledge about Lean, continuous development, and digitally available data. The factors that had hindered commitment to LM included organizational, environmental, and psychological factors and a lack of knowledge and data.

**Table 3.** Factors supporting and hindering commitment of LM

Supporting factors
(i) Organizational factors
(a) Leadership a tiered management system standardized meetings for units, divisions and sectors; meetings that have hybrid options multiprofessional cooperation
(ii) Psychological factors positive work atmosphere leaders’ own commitment to Lean and using LM daily staff commitment sensitive dialogue with staff and multiprofessional respect for each other staff see that things are getting better
(iii)Knowledge knowledge and education about LM
(iv) Developing Lean developing unit and educated Lean coaches Lean coaches have allocated time for development and support regular development meetings one development activity at a time
(v) Available data metrics that can be obtained digitally
Hindering factors
(i) Organizational factors
**(a)** Lack of resources physical premises are too old, small, and unsuitable for operations lack of employees in hospitals and an ever-changing staff staff must hurry because of patient overload not enough time and staff for development projects not enough operating rooms in use no usable results from development projects, which generates doubts about LM
(b) Organizational structure hierarchical organization
(c) Leadership fear that the developments will be focused elsewhere under a new general leader
(ii) Environmental factors ongoing social factors: coronavirus epidemic, war in Ukraine, nursing strike in Finland
(iii) Psychological factors unit leaders and staff are not committed former attitudes and beliefs undermine LM culture-bound thinking about work tasks have not been able to implement Lean thinking and operations in the units
(iv) Lack of knowledge low levels of education about LM
(v) Lack of data the necessary real-time functional metrics are not available from information systems the long-term monitoring of development is lacking, causing unit operations to revert back to the old model

The factors that support and hinder good communication in the three sectors are shown in Table 4. The supporting factors consist of psychological and organizational factors and knowledge about Lean and LM, while the hindering factors tended to negatively affect communication between people, namely, various environmental, organizational, and psychological factors.

**Table 4.** Factors supporting and hindering communication.

Supporting factors
(i) Psychological factors trust in other people open interactions genuine listening
(ii) Organizational factors
(a) Leadership common goals autonomous actions weekly Gemba walks daily and weekly meetings daily management boards active Lean thinking and use of LM
(iii) Knowledge knowledge and education regarding Lean and LM
Hindering factors
(i) Environmental factors ongoing social factors: coronavirus epidemic, war in Ukraine, nursing strike in Finland
(ii) Organizational factors
(a) Lack of resources lack of employees lack of time
(b) Information system the new electronic health record system is not working well
(c) Organizational structure hierarchical organization
(d) Psychological factors lack of coping with feelings on the part of leaders and staff lack of commitment to Lean and LM

## Discussion

### Statement of principal findings

Lean has a dynamic nature in the heterogeneous environment of health care that makes it difficult to adopt in practice. According to our findings, the organization-wide Lean implementation needs a good knowledge about Lean and correct leadership activities that align also with the findings of Hilverda *et al*. [22] on key instruments to increase the maturity of Lean. In hospitals, achieving the desired results with Lean requires a high level of maturity, which needs to be repeatedly evaluated and maintained for continuous improvement by using a low-threshold maturity tool.

Our findings relate to previous research illustrating similarities and extending earlier work [4, 20, 22, 25–27] that the knowledge about Lean, the availability of data, environmental, psychological, and organizational factors play important roles in supporting and hindering leadership, communication and daily management in the hospital, and commitment to Lean. According to our findings, leadership and a commitment to Lean are strongly interconnected. We agree with the previous findings [25, 28], when leaders have misunderstanding about Lean, it is related to mechanical implementation of Lean, which indicates a low maturity level of Lean in organizations.

Recent studies emphasized markers of transformation, the depth and breadth of Lean scoring scales [20], and the dimensions impacting the implementation of Lean together with its outcomes [18]. Here, our research adds descriptive knowledge via a dimensions-centric perspective assessing Lean maturity development. Our findings show that it is most important to focus on five dimensions and the explanatory factors as well as easy-to-use Lean maturity tools when evaluating and developing Lean implementation of organizations continuously.

### Strengths and limitations

The strength of this research is the consideration of the qualitative data on Lean maturity in a hospital setting. The study shows that to demonstrate the maturity of Lean in hospitals, descriptive data from the staff are needed to better understand what factors affect the implementation of Lean because numerical data alone are not sufficient.

The limitation of this research is that the interviews did not cover every leader or employee of the hospital, and it does not present the level of maturity in numerical terms. The number of participants was limited (*N* = 15) to one hospital, but sufficient because during the content analysis, the responses became saturated. The research included one researcher who interviewed all participants, so the researcher’s objectivity and knowledge about Lean should be considered with the findings, although all the research findings were verified among the group of researchers. For further research, it would be good to use quantitative research and complement it with qualitative research to obtain numerical data and descriptive information about Lean and establish a connection between Lean maturity and the performance of hospitals.

### Interpretation within the context of the wider literature

Recent studies on the use of Lean maturity tools highlight their statistical accuracy since they focus on the factors influencing Lean implementation and its maturity level [17–20, 23]. In contrast, this study describes the differences in maturity by dimension rather than by measures of LM maturity level. Based on our findings, the maturity level of Lean varied in different sectors within the same hospital; for example, the high maturity level in Sector B signifies that Lean had become part of the daily routine and makes it possible to implement a principle-driven form of Lean. Our findings provide descriptive information to leaders about the maturity of Lean in health care, giving them an opportunity to benchmark the information and benefit from it through continuous learning.

Our findings fit with the case studies by Charns *et al*. [20], concerning the enablers of Lean, and Leite *et al*. [26], concerning barriers to the organizational transformation of Lean. We extend this line of research by focusing on both the implementation of LM and its supporting and hindering factors and its maturity. Our study fits with the findings of a literature review by Kunnen *et al*. [21] on the facilitators in and barriers to LM. The similarities in the findings make this research transferable and relevant for evaluating LM in other healthcare contexts.

### Implications for policy, practice, and research

In Finland, there is healthcare legislation [29] that obligates hospitals to start the care of patients within a certain time, which causes pressure for leaders. We found that the adoption of Lean in the hospital aimed to improve internal efficiency and was intermixed with other external production pressures. This finding is the one of environmental differences in Finnish health care compared to international research findings. We agree with the studies by D’Andreamatteo *et al*. [4] and Leite *et al*. [26], who suggest that policymakers and leaders should understand Lean as a framework that promotes patient’s needs and the staff’s well-being. Our findings confirm that the hospital in Finland has faced many challenges, similar to other hospitals in Canada [14] and the UK [30], without being able to do Lean transition into hospital-level cultural change. Therefore, hospitals that are not fully committed to Lean cannot reach their goals and full potential [14, 31].

LM offers a structured model for assessing the resources being wasted and for resolving problems through cooperation [2, 27, 31]. In our study, standardized work increased the safety and quality of care, strengthened the employees’ competence, and thus increased their flexibility to tasks where they were most needed. The flexibility of the staff reduced the negative impact from a lack of employees, which plagued the hospital. Previous studies found that effective patient care processes decrease the costs to patients and their municipalities [2, 27, 31]. However, numerical data on the costs were not evaluated in this research. Future research should address the effects of the maturity of Lean on healthcare outcomes and costs.

Our maturity evaluation method proved to be easier to use than earlier methods and required less work in relation to the evaluation process. In comparison, in the research work of Charns *et al*. [20], 6 researchers visited 10 medical centers 3 times each, while interviewing 121 leaders, taking notes and reviewing the documents. Charns *et al*. [20] also used markers of Lean transformation that scored its breadth and depth, creating the enablers of Lean transformation. Nevertheless, we noticed that during our interviews, the responses became saturated, and we obtained enough answers to assess the maturity of Lean. We noticed that focusing on key dimensions of LHISI provides insight into the maturity of Lean and the factors influencing it.

## Conclusion

This study shows that LM is a comprehensive management system and its technical implementation alone is not enough. The main theoretical findings are that the organization-wide implementation requires a good knowledge of Lean and correct leadership activities that also increase the maturity level of it. By developing and maintaining the key dimensions of Lean, organizations can continuously develop and achieve their full potential. The main practical conclusion of our study is that the Lean maturity tools should be easy to use, so organizations themselves can evaluate information about Lean implementation and the factors that influence it.

## Data Availability

Data are available only on request.

## Permission

HUS Helsinki University Hospital.

## Appendix

### Attachment 1

The interview questions according to LHISI maturity model.

### Resource:

Reponen E. *et al*. Validation of the Lean Healthcare Implementation Self-Assessment Instrument (LHISI) in the Finnish healthcare context. *BMC Health Serv Res* (2021); **21**:1289

### 1. Leadership

- Describe in your own words what Lean management means?
- How is Lean management used in your sector/division/unit? Provide examples.
- How is Lean management used in your work? Provide examples.
- Evaluate how much Lean management is used in your work daily/weekly/monthly, etc.

### 2. Commitment

- What Lean development tools (if any) are used in your sector/division/unit?
- Do you think there is continuous improvement in your sector/division/unit?
  - If so, describe it.
- Has anything helped or hindered the development of Lean in your sector/division/unit?
- Evaluate how involved you and the co-workers in your sector/division/unit are with the Lean ideology.

### 3. Standard work

- Describe how the work has been standardized in your sector/division/unit.
- Evaluate how Lean has streamlined processes in your sector/division/unit.

### 4. Communication

- How are current and developing topics communicated to you or by you in your sector/division/unit? Are there any recurring meetings regarding these topics, and who is present in them?
- Has Lean made communications clearer in your sector/division/unit?
- Evaluate how well the employees know your common goals and if you are all working toward them.

### 5. Daily management system

- Evaluate how much Lean is used daily in your sector/division/unit every week or month.
- How is Lean used in leadership daily in your sector/division/unit?
- Are Lean tools, such as daily managing systems, Gemba-walking, description of the processes, and 5S, used in your sector/division/unit?
- Is A3 used in your sector/division/unit?
- Evaluate the applicability of Lean in daily management in your sector/division/unit.

### 6. Lean management evaluation of effectiveness

- How long has Lean management been implemented in your sector/division/unit?
- Has Lean been useful for meeting the needs of your sector/division/unit?
- Describe in your own words what has been achieved with Lean management in your sector/division/unit.
- Has Lean management led to any desired outcomes in your sector/division/unit? If so, describe them.
- Have any Lean projects been abandoned or ceased? If so, why?
- Describe the currently ongoing Lean projects in your sector/division/unit? What are their goals?
- Are there any other development processes that have better suited your sector/division/unit? Describe them and their effects.
- Evaluate how the patients have benefited from Lean development.
- Evaluate Lean management in your sector/division/unit and how it can be applied to other special health care services.
